# Abdominal wall hernias in abdominal aneurysm cases: a cross-sectional imaging study

**DOI:** 10.31744/einstein_journal/2026AO2405

**Published:** 2026-08-12

**Authors:** Nathalia Cardoso Oliveira, Maria Fernanda Portugal, Matheus Toledo Nora, Paula Ribeiro do Prado Chadud, Luciana Helena Benetti, Camila de Freitas Corrêa, Adriano Tachibana, Ricardo Aun, Sergio Quilici Belczak

**Affiliations:** 1 Instituto Maravasc São Luís MA Brazil Instituto Maravasc, São Luís, MA, Brazil.; 2 Hospital Israelita Albert Einstein São Paulo SP Brazil Hospital Israelita Albert Einstein, São Paulo, SP, Brazil.; 3 Instituto de Aprimoramento e Pesquisa em Angiorradiologia e Cirurgia Endovascular São Paulo SP Brazil Instituto de Aprimoramento e Pesquisa em Angiorradiologia e Cirurgia Endovascular, São Paulo, SP, Brazil.; 4 Universidade de São Paulo Department of Vascular and Endovascular Surgery São Paulo SP Brazil Department of Vascular and Endovascular Surgery, Universidade de São Paulo, São Paulo, SP, Brazil.

**Keywords:** Hernia, abdominal, Hernia, hiatal, Aneurysm, Aortic aneurysm, abdominal, Imaging diagnostic

## Abstract

**Objective:**

This study aimed to assess the prevalence of abdominal and hiatal hernias in patients with aortic, visceral, and renal aneurysms in a quaternary hospital.

**Methods:**

Computed tomography angiography reports from 2,597 patients with intra-abdominal aneurysms, collected between January 2005 and July 2021 at a private hospital in São Paulo, Brazil, were analyzed. The variables of interest were analyzed descriptively, and inferential statistics (chi-square test and Student's *t*-test) were applied to explore relationships between sex, age, number of vessels with aneurysms, and number of hernias identified by computed tomography angiography.

**Results:**

Of 2,597 patients with abdominal aneurysms, 837 (32.23%) presented with at least one hernia. Among them, 416 patients (16.03%) had abdominal wall hernias, while 421 (16.20%) had hiatal hernias. Patients with hernias were significantly older (72.2±12.0 years; p<0.001) than patients without hernias (68.2±14.0 years); there were no significant age differences between sexes (p=0.714). Hiatal hernias had a high prevalence (50.3%) among both aortic and visceral aneurysms, particularly amnong those with hepatic artery (42.91%) and celiac trunk aneurysms (41.82%).

**Conclusion:**

A high coexistence of abdominal and hiatal hernias was observed among patients with intra-abdominal aneurysms. These findings highlight a statistical association and should not be interpreted as indicating a causal relationship or as a risk-stratification tool in the absence of prospective, controlled studies.

## Highlights

A total of 32.23% of patients with aneurysms had abdominal or hiatal hernias.

Hiatal hernia was the most frequent associated finding.

Highest frequencies occurred in hepatic and celiac aneurysms.

Findings support future prospective studies.

## In Brief

In this cross-sectional imaging study, patients with intra-abdominal aneurysms showed a high frequency of abdominal and hiatal hernias (32.23%). Hiatal hernias were the most frequent finding. Higher hernia frequencies were observed in patients with hepatic and celiac trunk aneurysms. The findings are descriptive and hypothesis-generating.

## Graphical abstract

**Figure:**
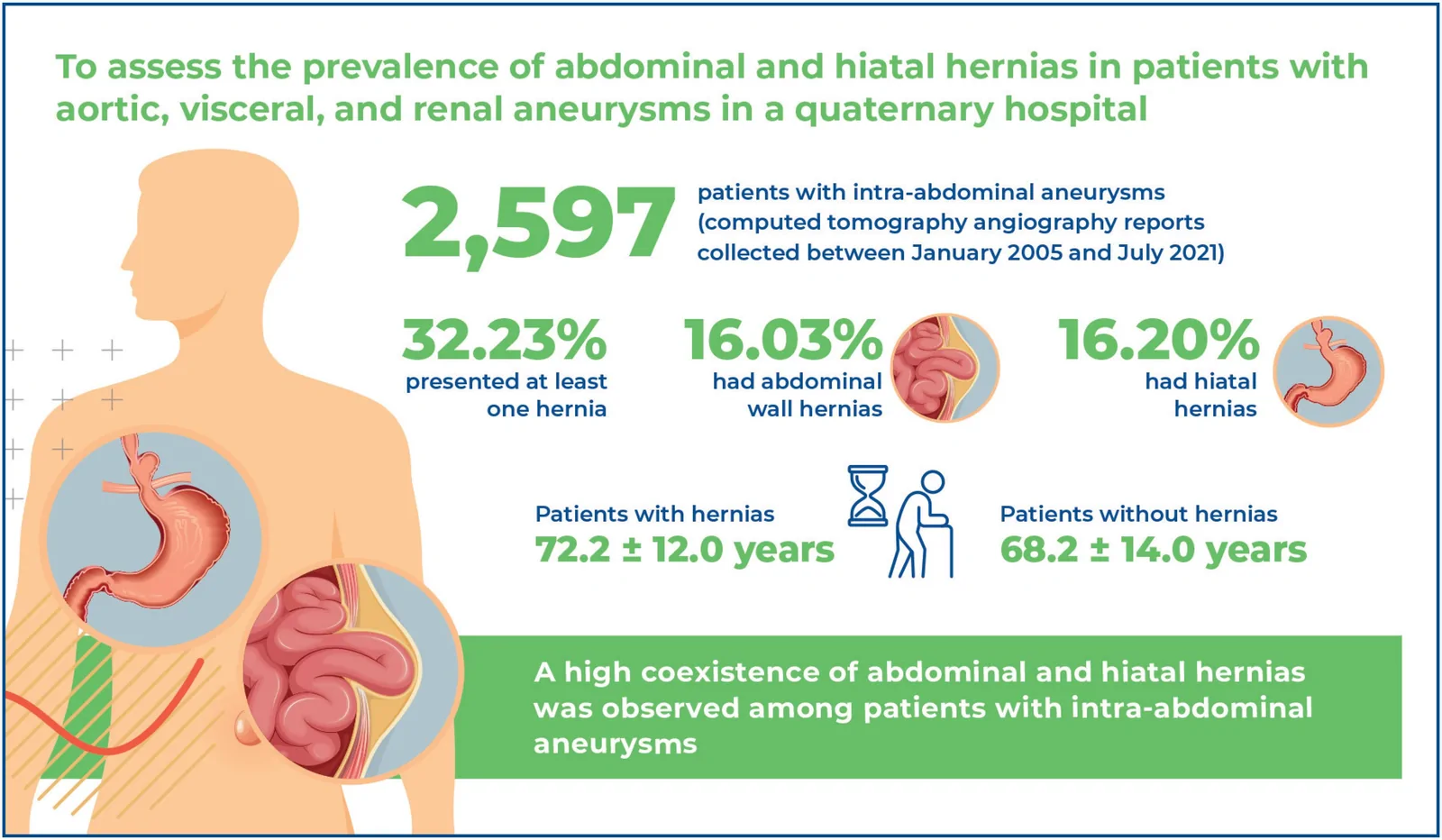

## INTRODUCTION

Several studies have found a strong association between intra-abdominal aneurysms (IAA) and abdominal hernias, particularly between abdominal aortic aneurysms (AAA) and abdominal wall hernias (AWH).^(1-5)^ In a meta-analysis of 14 studies (including 39,262 patients), a 2.3 times higher incidence of AAA in patients with AWH was found.^(6)^ Kontogeorgi et al. confirmed the high probability that patients with AWH would develop AAA, even recommending that AWH should be used as a criterion in AAA screening in addition to age, sex, family history, and smoking.^(7)^ Hung et al. found an association between AWH, AAA, and thoracoabdominal aneurysms.^(5)^

Nonetheless, the relationship between aneurysms and hernias remains multifactorial and not fully understood. Beyond possible connective tissue alterations, several epidemiological and environmental factors, such as age, smoking, atherosclerosis, and increased intra-abdominal pressure, are likely to play concurrent roles in the development of both conditions.^(2,8-12)^

Important studies have focused on the association between inguinal hernias, AAA, and thoracoabdominal aneurysms, although the findings of some studies were negative.^(3,13-16)^ In contrast, few investigations have extended this analysis to include other types of abdominal wall or diaphragmatic hernias, such as hiatal hernias (HH), or to aneurysms located in visceral and renal arteries. This knowledge gap limits our understanding of whether the coexistence observed in AAA also applies to aneurysms in other abdominal territories.

In this context, and recognizing the limitations of retrospective imaging-based designs, this study aimed to describe and characterize the pattern of distribution of AWH and HH in patients with IAA, including aortic, visceral, and renal locations, identified through computed tomography angiography (CTA) performed at a tertiary referral center.

Rather than testing a causal relationship, this study sought to delineate the frequency and anatomical distribution of different hernia types in this specific population, contributing to the descriptive mapping of this clinically relevant association.

## OBJECTIVE

This study aimed to describe and characterize the pattern of distribution of abdominal wall hernias and hiatal hernias in patients with intra-abdominal aneurysms, including aortic, visceral, and renal locations, identified through computed tomography angiography performed at a tertiary referral center.

## METHODS

This is a retrospective, cross-sectional study based on CTA reports of patients diagnosed with IAA. All data were collected from the radiological information system of a private quaternary hospital in São Paulo, Brazil, between January 2005 and July 2021. This study was carried out in accordance with the Code of Ethics of the World Medical Association (Declaration of Helsinki). The study protocol was approved by the Ethics Committee of the *Hospital Israelita Albert Einstein* (CAAE: 47103021.0.0000.0071; # 5,081,688), and the requirement for informed consent was waived due to the retrospective nature of the research.

### Study population and inclusion criteria

All CTA examinations reporting the presence of an intra-abdominal aneurysm, defined as a true aneurysm in any abdominal vessel (aorta, visceral, or renal arteries), were included. Only the first examination per patient was considered; subsequent follow-up or duplicate scans were excluded to avoid duplicate data from the same individual. No additional exclusion criteria were applied, as image quality and reporting consistency were ensured by the high technical standards of the institution. Because the study focused on the presence and distribution of aneurysms rather than their dimensions, no size or measurement thresholds were required beyond the definition described below.

### Diagnostic criteria and data extraction

Aneurysms were defined as vessels with a diameter at least 1.5 times greater than the normal adjacent segment, as determined by the radiologist using the centering ruler on CTA. Hernias were identified based solely on explicit descriptions present in the finalized radiology reports; no manual re-evaluation of images was performed. The dataset included the following variables: patient identification number, sex, age, date of examination, affected arteries, and type and location of any abdominal or hiatal hernia.

### Data validation

To ensure consistency and reliability, all extracted data were independently verified through a double-checking process by two experienced researchers. One of the investigators was a board-certified radiologist who confirmed the methodological accuracy of the data extraction and classification procedures.

### Statistical analysis

Statistical analyses were performed using standard descriptive and bivariate methods. Categorical variables were summarized as absolute and relative frequencies, and continuous variables as means and standard deviations. Comparisons between groups were made using the χ^2^ test for categorical variables and Student's t-test for continuous variables. Pearson's correlation coefficient was used to explore relationships between the number of aneurysmal vessels and the number of hernia locations identified by CTA. A p<0.05 was considered statistically significant.

Given the retrospective and exploratory nature of this study and the absence of a control group, multivariable analysis was not applied. The aim of the analysis was to describe the distribution and frequency of hernias among patients with IAA rather than to infer causality or adjust for confounding variables. Furthermore, because hernias were identified exclusively from finalized radiology reports without direct re-evaluation of the CTA images, the reported prevalence may underestimate the true prevalence, particularly for small or asymptomatic hernias that may not have been explicitly described in the original reports.

## RESULTS

Among 141,836 CTA scans performed between January 2005 and July 2021, 48,953 (34.5%) were excluded for being follow-up or duplicate exams from the same patients. The remaining 92,883 unique scans identified 2,597 patients with IAA, who constituted the final study cohort.

Of the 2,597 patients with IAA, 837 (32.2%) presented with at least one abdominal or hiatal hernia (Table 1). The overall prevalence of hernias did not differ significantly between sexes (32.4% in women versus 32.1% in men; p=0.876). Patients with hernias were significantly older than those without hernias (mean age 72.2±12.0 versus 68.2±14.0 years; p<0.001).

**Table 1:** Distribution of patients with intra-abdominal aneurysms according to the presence of abdominal hernias by sex and age (in years)

Abdominal hernias	Female n (%)	Male n (%)	Total n (%)	Age (mean±standard deviation)		
				Female	Male	Total
Present	327 (32.40)	510 (32.11)	837 (32.23)	72.4±13.3	72.1±10.9	72.2±12.0
Absent	682 (67.60)	1,078 (67.88)	1,760 (67.77)	66.5±15.5	69.2±12.9	68.2±14.0
Total	1,009 (100.0)	1,588 (100.0)	2,597 (100.0)			
P value	0.876	0.876	0.876	<0.001	<0.001	<0.001

SD: standard deviation.

HH was the most frequent type (50.3%), followed by umbilical (27.0%) and inguinal hernias (right: 16.9%; left: 14.3%). HH were significantly more common among women (66.4%, p<0.001), whereas inguinal and epigastric hernias predominated in men (p<0.001 and p=0.031, respectively). The frequencies of other hernia types, paraumbilical, incisional, Spiegelian, and femoral, were low and showed no sex difference (Table 1S, Supplementary Material).

Patients with inguinal hernias were generally older than those with other hernia types, and women with inguinal hernias were significantly older than men (mean age 82.5±6.4 *versus* 73.3±9.9 years; p<0.01). For all other hernia types, no significant age difference between sexes was observed (Table 2S, Supplementary Material).

Most patients (76.8%) had a single hernia type, while 23.2% had multiple hernias (Figure 1). The most frequent concomitance occurred between right and left inguinal hernias, indicating frequent bilaterality. The second most common association was between hiatal and umbilical hernias. Other less frequent combinations included paraumbilical with umbilical hernias and spigelian with inguinal hernias (Figure 1S, Supplementary Material).

**Figure 1:**
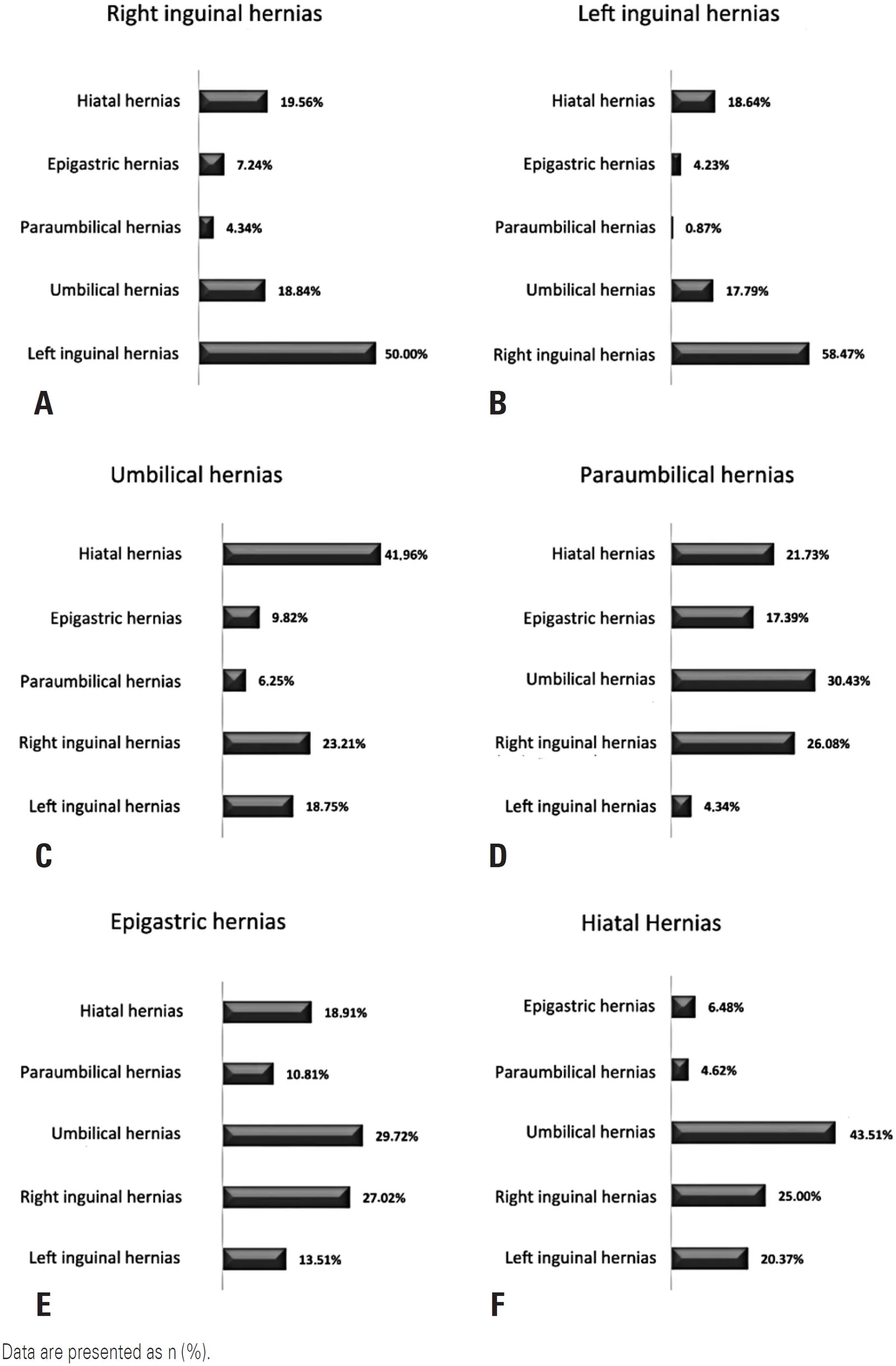
Concomitance between different types of abdominal hernias. (A) Right inguinal hernias (n=101); (B) Left inguinal hernias (n=89); (C) Umbilical hernias (n=84); (D) Paraumbilical hernias (n=19); (E) Epigastric hernias (n=25); (F) Hiatal hernias (n=84)

The frequency of hernias according to the aneurysmal artery followed a similar distribution to the overall cohort, except for the hepatic artery and the celiac trunk aneurysms, which showed a higher prevalence of hernias (42.9% and 41.8%, respectively) (Figure 2S, Supplementary Material). HH were particularly common in patients with aneurysms of the hepatic, splenic, and left renal arteries. No significant correlation was found between the number of aneurysmal vessels and the number of hernia locations (r=0.02). A non-significant positive correlation was observed between age and the number of hernia sites (r=0.12).

## DISCUSSION

In this large retrospective cohort, approximately one-third of patients with IAA also presented with abdominal or HH. This prevalence is notably higher than that reported in the general population, suggesting that patients with aneurysmal disease may have a greater predisposition to hernia formation. However, these results should be interpreted with caution, as this study was designed to describe coexistence patterns rather than to infer causal relationships.

The frequencies of AWH (16.0%) and HH (16.2%) found in this cohort were considerably higher than those reported for the general population, in which the prevalence of AWH is estimated at 1.7–4% and of HH at 2–10%, depending on age and clinical condition.^(17,18)^ Previous studies have also demonstrated that the coexistence of AAA and hernias is common, with meta-analyses reporting a two- to threefold higher incidence of aneurysm in patients with hernias and vice versa.^(3,6,7)^ Our findings confirm this trend and expand the current evidence by including aneurysms of visceral arteries, a domain for which data remain limited.^(5,11,12)^

Consistent with previous reports, inguinal and umbilical hernias were the most frequent AWH among patients with aneurysms.^(17,19-21)^ We also identified a remarkable proportion of HH, particularly among patients with hepatic, celiac trunk, and splenic artery aneurysms, suggesting a specific pattern of coexistence that deserves further investigation.

The pathophysiological mechanisms underlying this coexistence remain uncertain. Current evidence suggests that both aneurysms and hernias may share alterations in connective tissue metabolism, involving imbalances in collagen synthesis and degradation and overexpression of matrix metalloproteinases.^(1,8-10,13,22,23)^ These enzymes weaken the extracellular matrix and may affect both vascular and fascial integrity. In addition, environmental and systemic factors such as smoking, atherosclerosis, and aging likely contribute to their simultaneous occurrence.^(2,11,12)^

Although our findings cannot establish causality, they emphasize that hernias are common among patients with aortic and visceral aneurysms, and that their coexistence may be more frequent than previously recognized. Awareness of this association may have clinical relevance, as the detection of a hernia in an older patient with vascular risk factors could prompt evaluation for possible aneurysmal disease. Similarly, the presence of hiatal or umbilical hernias in patients with known aneurysms may support a more comprehensive surgical and radiological approach.

This study had some limitations. It was based on a retrospective sample of convenience derived from existing CTA reports, without inclusion of a control group. Direct comparison with the general population was therefore not possible. Minor, reducible, or asymptomatic hernias may have been underreported, since data were obtained from finalized radiology reports without manual re-evaluation of images. The study population also represents a quaternary center, which may have led to selection bias toward more complex cases. Furthermore, demographic and clinical variables such as smoking, body mass index, or comorbidities were unavailable, precluding adjustment for potential confounders.

Despite these limitations, this study provides one of the largest descriptive analyses to date of hernia occurrence among patients with IAA, encompassing both aortic and visceral topographies. The high frequency of hiatal, umbilical, and inguinal hernias underscores the need for future prospective studies to confirm these findings and to clarify the biological and clinical mechanisms underlying this coexistence.^(24)^

An ideal next step would be a prospective, multicenter case-control study including patients with and without IAA, with systematic and standardized radiological evaluation for hernias, detailed clinical data (including smoking status, body mass index, and comorbidities), and assessment of biochemical markers related to connective tissue remodeling, such as matrix metalloproteinases. Such a design would allow adjustment for confounding variables, more accurate prevalence estimates, and better exploration of potential shared pathophysiological mechanisms.

## CONCLUSION

This study demonstrated a high frequency of abdominal and hiatal hernias among patients with intra-abdominal aneurysms, including both aortic and visceral topographies. The most frequent hernia types were hiatal, umbilical, and inguinal, showing a recognizable pattern of coexistence within this population.

Given the retrospective and descriptive design, these findings should be interpreted as hypothesis-generating rather than causal. These findings highlight a statistical association and should not be interpreted as indicating a causal relationship or as a risk-stratification tool in the absence of prospective, controlled studies. Nevertheless, they provide relevant insights into the distribution of hernias among patients with aneurysmal disease and highlight the need for prospective studies to further explore the biological and clinical mechanisms behind this association.

## Data Availability

The underlying content is contained within the manuscript.

## References

[1] Antoszewska M (2013). Evaluation of the levels of metalloproteinsase-2 in patients with abdominal aneurysm and abdominal hernias. Pol Przegl Chir.

[2] Antoniou GA, Georgiadis GS, Antoniou SA, Granderath FA, Giannoukas AD, Lazarides MK (2011). Abdominal aortic aneurysm and abdominal wall hernia as manifestations of a connective tissue disorder. J Vasc Surg.

[3] Antoniou GA, Giannoukas AD, Georgiadis GS, Antoniou SA, Simopoulos C, Prassopoulos P (2011). Increased prevalence of abdominal aortic aneurysm in patients undergoing inguinal hernia repair compared with patients without hernia receiving aneurysm screening. J Vasc Surg.

[4] Gindera LB, Donas KP, Torsello G, Bisdas T, Stavroulakis K (2015). Significant CT-angiographic evidence of coexisting abdominal aortoiliac aneurysms with simple renal cysts and abdominal wall hernias. Minerva Chir.

[5] Hung KC, Chang YJ, Sun CK, Wang JJ, Chen YC, Weng SF (2021). Association of hernia with subsequent aortic aneurysm in geriatric patients. J Thorac Cardiovasc Surg.

[6] Takagi H, Umemoto T (2015). A meta-analysis of the association of primary abdominal wall hernia with abdominal aortic aneurysm. Int Angiol.

[7] Kontogeorgi E, Sagris M, Kokkinidis DG, Hasemaki N, Tsakotos G, Tsapralis D (2021). Abdominal aortic aneurysms and abdominal wall hernias - a systematic review and meta-analysis. Vasa.

[8] Jansen PL, Mertens Pr P, Klinge U, Schumpelick V (2004). The biology of hernia formation. Surgery.

[9] Klinge U, Binnebösel M, Mertens PR (2006). Are collagens the culprits in the development of incisional and inguinal hernia disease?. Hernia.

[10] Antoniou GA, Tentes IK, Antoniou SA, Georgiadis GS, Giannoukas AD, Simopoulos C (2011). Circulating matrix metalloproteinases and their inhibitors in inguinal hernia and abdominal aortic aneurysm. Int Angiol.

[11] Pitoulias GA, Donas KP, Chatzimavroudis G, Torsello G, Papadimitriou DK, Simopoulos C (2012). The role of simple renal cysts, abdominal wall hernia, and chronic obstructive pulmonary disease as predictive factors for aortoiliac aneurysmatic disease. World J Surg.

[12] Pannu R, McPhail IR (2012). Prevalence of abdominal wall hernia in participants with abdominal aortic aneurysm versus peripheral arterial disease—a population-based study. Angiology.

[13] Pleumeekers HJ, De Gruijl A, Hofman A, Van Beek AJ, Hoes AW (1999). Prevalence of aortic aneurysm in men with a history of inguinal hernia repair. Br J Surg.

[14] Megalopoulos A, Ioannidis O, Varnalidis I, Ntoumpara M, Tsigriki L, Alexandris K (2019). High prevalence of abdominal aortic aneurysm in patients with inguinal hernia. Biomed Pap Med Fac Univ Palacky Olomouc Czech Repub.

[15] Olsson C, Eriksson P, Franco-Cereceda A (2014). Association between thoracic aortic disease and inguinal hernia. J Am Heart Assoc.

[16] Henriksen NA, Sorensen LT, Jorgensen LN, Lindholt JS (2013). Lack of association between inguinal hernia and abdominal aortic aneurysm in a population-based male cohort. Br J Surg.

[17] Dabbas N, Adams K, Pearson K, Royle G (2011). Frequency of abdominal wall hernias: is classical teaching out of date?. JRSM Short Rep.

[18] Kim J, Hiura GT, Oelsner EC, Yin X, Barr RG, Smith BM (2021). Hiatal hernia prevalence and natural history on non-contrast CT in the Multi-Ethnic Study of Atherosclerosis (MESA). BMJ Open Gastroenterol.

[19] Kingsnorth A, LeBlanc K (2003). Hernias: inguinal and incisional. Lancet.

[20] Wang R, Qi X, Peng Y, Deng H, Li J, Ning Z (2016). Association of umbilical hernia with volume of ascites in liver cirrhosis: a retrospective observational study. J Evid Based Med.

[21] Ponten JE, Somers KY, Nienhuijs SW (2012). Pathogenesis of the epigastric hernia. Hernia.

[22] Antoniou SA, Antoniou GA, Granderath FA, Simopoulos C (2009). The role of matrix metalloproteinases in the pathogenesis of abdominal wall hernias. Eur J Clin Invest.

[23] Georgiadis GS, Antoniou GA, Argyriou C, Schoretsanitis N, Nikolopoulos E, Kapoulas K (2019). Correlation of Baseline Plasma and Inguinal Connective Tissue Metalloproteinases and Their Inhibitors With Late High-Pressure Endoleak After Endovascular Aneurysm Repair: long-term Results. J Endovasc Ther.

[24] Isola D, Jawad Z, Chua E, Shorvon P, Martinez-Isla A (2022). Traction-induced aneurysm of an accessory left hepatic artery: a potentially fatal but synchronously treatable complication of a large hiatus hernia. Ann R Coll Surg Engl.

