# Supplementary material for: Abdominal wall hernias in abdominal aneurysm cases: a cross-sectional imaging study
Source: Einstein (Sao Paulo). 2026 Aug 12;24:eAO2405. doi: 10.31744/einstein_journal/2026AO2405 (PMC13682778; doi:10.31744/einstein_journal/2026AO2405)
Supplement: Supplementary file 1 [file 2317-6385-eins-24-eAO2405-Suppl01.pdf]

SUPPLEMENTARY MATERIAL

Abdominal wall hernias in abdominal aneurysm cases: a cross-sectional imaging study

Nathalia Cardoso Oliveira, Maria Fernanda Portugal, Matheus Toledo Nora, Paula Ribeiro do Prado Chadud, Luciana Helena Benetti, Camila de Freitas Corrêa, Adriano Tachibana, Ricardo Aun, Sergio Quilici Belczak

DOI: 10.31744/einstein_journal/2026AO2405

Table 1S. Distribution of women and men according to the type of abdominal hernia

Abdominal hernias	Female (n=327) n (%)	Male (n=510) n (%)	Total (n=837) n (%)	p value
Right inguinal	9 (2.76)	132 (25.88)	141 (16.85)	<0.001
Left inguinal	8 (2.45)	112 (21.96)	120 (14.34)	<0.001
Umbilical	88 (26.91)	138 (27.06)	226 (27.00)	0.962
Paraumbilical	16 (4.89)	20 (3.92)	36 (4.30)	0.499
Epigastric	13 (3.98)	39 (7.65)	52 (6.21)	0.031
Spiegel right	5 (1.53)	3 (0.59)	8 (0.96)	0.172
Spiegel left	2 (0.61)	4 (0.78)	6 (0.72)	0.772
Incisional	15 (4.59)	32 (6.27)	47 (5.62)	0.300
Right femoral	1 (0.31)	1 (0.20)	2 (0.24)	0.751
Left femoral	1 (0.31)	1 (0.20)	2 (0.24)	0.751
Hiatal	217 (66.40)	204 (40.0)	421 (50.30)	<0.001

Table 2S. Means and standard deviations of the ages of women and men according to the location of abdominal hernias

Abdominal hernias	Female	Male	Total	p value
Right inguinal	82.9±5.58	72.9±10.5	73.6±10.5	0.005
Left inguinal	82.0±7.5	73.8±9.3	74.3±9.4	0.016
Umbilical	67.2±15.4	67.8±9.7	67.6±12.2	0.366
Paraumbilical	72.2±10.5	69.4±13.9	71.1±12.5	0.158
Epigastric	68.3±12.2	76.6±11.2	72.2±11.5	0.324
Incisional	69.7±15.0	70.2±10.8	70.1±12.1	0.899
Hiatal	74.8±12.2	73.9±11.2	74.4±11.7	0.481

Due to the small number of cases, we did not include femoral and Spiegel-type hernias in this analysis.

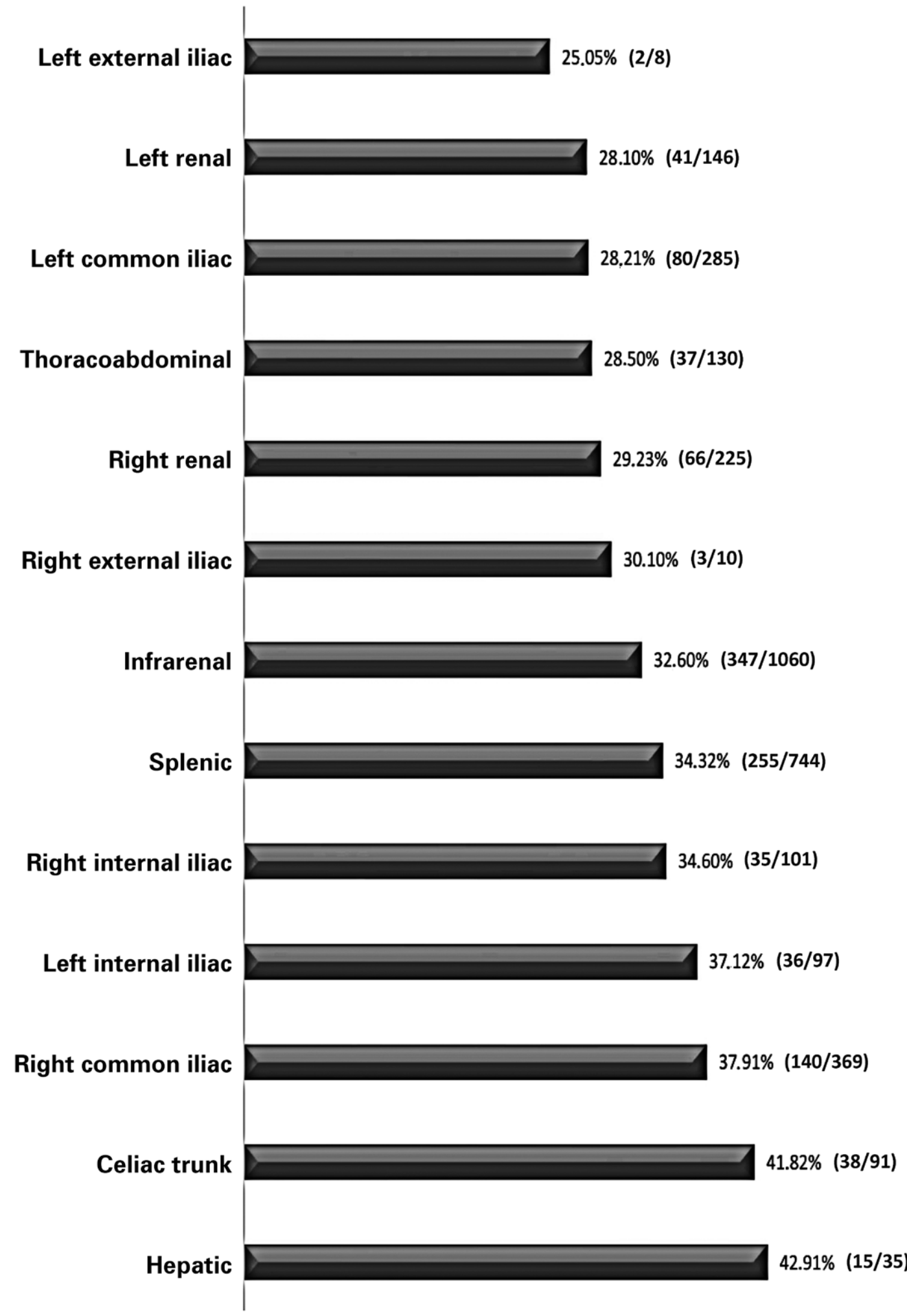

Data are presented as n (%).

Figure 1S. Distribution of abdominal hernias in relation to arterial aneurysms

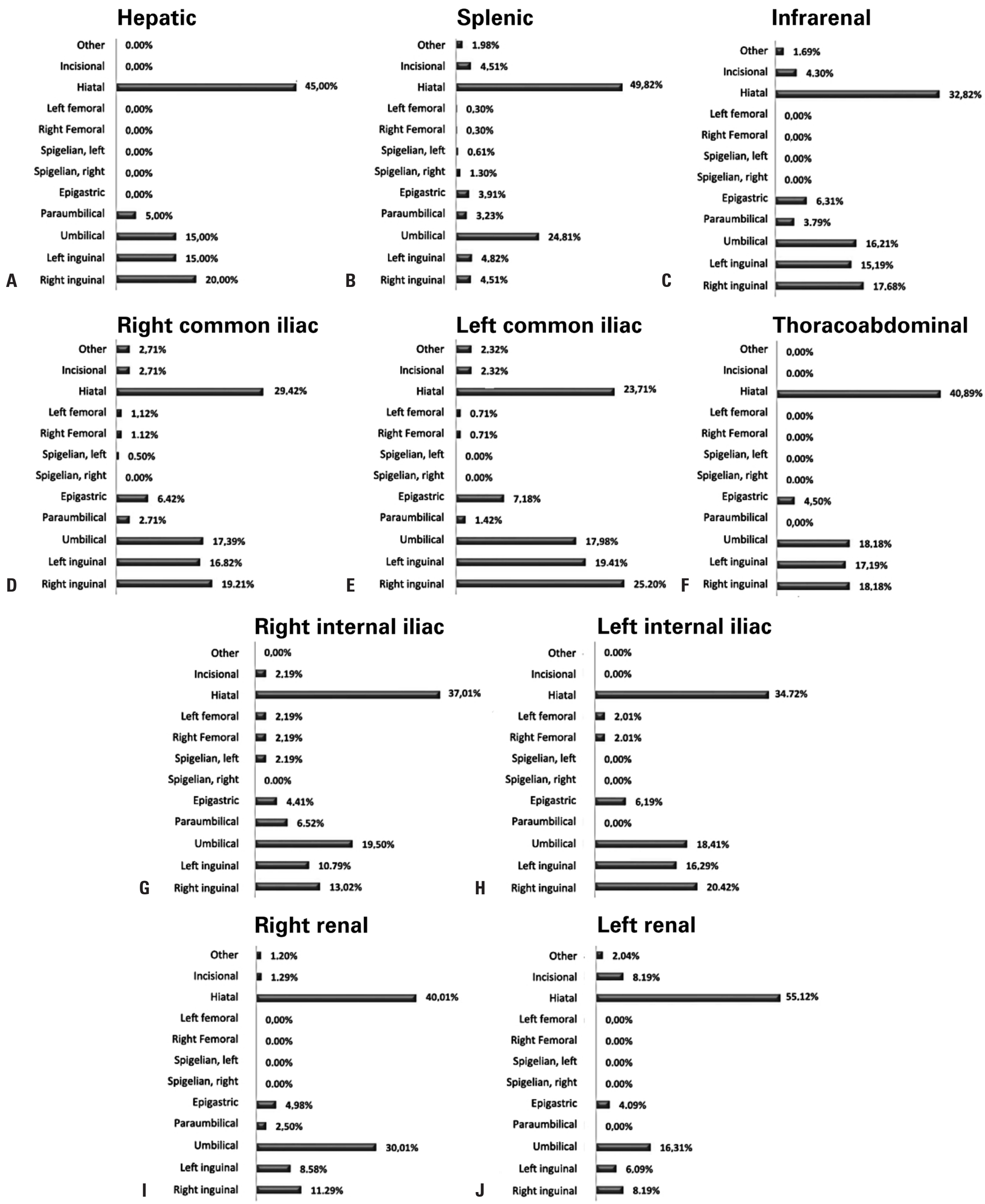

Data are presented as n (%).

Figure 2S. Concomitance of abdominal hernias with arterial aneurysms. (A) Hepatic artery (n=359); (B) Splenic artery (n=287); (C). Infrarenal aorta (n=273); (D) Right common iliac artery (n=317); (E). Left common iliac artery (n=236); (F) Thoracoabdominal aorta (n=238); (G). Right internal iliac artery (n=289); (H) Left internal iliac artery (n=310); (I) Right renal artery (n=244); (J) Left renal artery (n=235)
